# Self-Harm in Eating Disorders (SHINE): a mixed-methods exploratory study

**DOI:** 10.1136/bmjopen-2022-065065

**Published:** 2022-07-27

**Authors:** Anna Lavis, Sheryllin McNeil, Helen Bould, Anthony Winston, Kalen Reid, Christina L Easter, Rosina Pendrous, Maria Michail

**Affiliations:** 1 Institute of Applied Health Research, University of Birmingham, Birmingham, UK; 2 Forward Thinking Birmingham, Birmingham Women’s and Children’s NHS Foundation Trust, Birmingham, UK; 3 Centre for Academic Mental Health, Population Health Sciences, Bristol Medical School & Medical Research Council Integrative Epidemiology Unit, University of Bristol, Bristol, UK; 4 Gloucestershire Health and Care NHS Foundation Trust, Gloucester, UK; 5 Coventry and Warwickshire Partnership Trust, Coventry, UK; 6 Youth Advisory Group, Institute for Mental Health, University of Birmingham, Birmingham, UK; 7 Institute for Mental Health, University of Birmingham, Birmingham, UK

**Keywords:** eating disorders, suicide & self-harm, child & adolescent psychiatry

## Abstract

**Introduction:**

Self-harm is highly prevalent among young people with eating disorders. However, why a young person may develop and continue to experience both an eating disorder and self-harm is unclear. This study will investigate the frequency, intensity, duration, function, context and processes of self-harm among people aged 16–25 diagnosed with an eating disorder. It will explore participants’ perspectives on the genesis and functions of both their self-harm and eating disorder, as well as their support needs. The study was designed with the input of members of a Young Persons’ Advisory Group, who will be key to study delivery and dissemination.

**Methods and analysis:**

This exploratory study has a sequential mixed-methods explanatory design. Between 70 and 100 young people aged 16–25 with both an eating disorder diagnosis and self-harm thoughts and/or behaviours will be recruited from three NHS Eating Disorder outpatient services in England. Phase 1: a 14-day (six prompts per day) ecological momentary assessment (EMA) of participants’ feelings, thoughts, motivations, behaviours and experiences of self-harm. Phase 2: 20–30 participants from phase 1 will be reapproached to take part in an in-depth qualitative interview on the psychological, emotional and social factors that underlie their self-harm and eating disorder as well as their support needs. EMA data from phase 1 will be analysed using descriptive and multilevel statistics. Qualitative interview data from phase 2 will be analysed using inductive and deductive thematic analysis. Results from both phases will be integrated using a mixed-methods matrix, with each participant’s data from both phases compared alongside comparative analysis of the datasets as a whole.

**Ethics and dissemination:**

The study gained ethical approval from the NHS HRA West Midlands–Black Country Research Ethics Committee (number: 296032). We anticipate disseminating findings to clinical, academic and lived experience audiences, at academic conferences, through peer-reviewed articles, and through various public engagement activities (eg, infographics, podcasts).

**Strengths and limitations of this study**

- A strength of this study is the mixed-methods design, integrating results from an ecological momentary assessment (EMA) study and a qualitative interview study.
- A further strength is that the study was designed with young people with lived experience and their continued involvement throughout will be key to delivering the study and disseminating results.
- A limitation of the EMA study is the lack of real-time assessment of ED-specific symptoms (eg, a co-occurring binge/purging episode).
- A limitation of the qualitative study is that interview participants will be looking back at the development of their self-harm and eating disorder and so accounts may be subject to recall bias.

## Introduction

This study will investigate the psychological, emotional and social factors that underlie self-harm (SH) in young people aged 16–25 with an eating disorder (ED). For this study we will use the National Institute for Health and Care Excellence (NICE) definition of SH, which refers to ‘any act of self-poisoning or self-injury carried out by an individual irrespective of motivation’ (2011, p4)^1^

A recent systematic review reported that EDs have an estimated lifetime prevalence (using weighed mean ranges) of 1.4% (0.1% to 3.6%) in women and 0.2% (0% to 0.3%) in men for anorexia nervosa; 1.9% (0.3% to 4.6%) in women and 0.6% (0.1% to 1.3%) in men for bulimia nervosa; 2.8% (0.6% to 5.8%) in women and 1.0% (0.3% to 2.0%) in men for binge ED; and 4.3% (0.6%–14.6%) in women and 3.6% (0.3%–5.0%) in men for ED not otherwise specified.^2^ Typically, EDs have a peak age of onset in adolescence^3^ and they result in major long-term burdens in terms of both morbidity and mortality,^4 5^ with high rates of relapse.^6^ Recent literature suggests that fewer than half of individuals with anorexia nervosa or bulimia nervosa fully recover.^7^

It is well-established that rates of SH in young people diagnosed with an ED are exceptionally high,^8 9^ ranging between 25.4% and 55.2%^10^ contrasting to 13.4% in the general population.^11^ Koutek *et al*’s study reported SH in 49% of their female participants with ED, and suicidal behaviour in 60%.^12^ SH is known to be one of the strongest risk factors for death by suicide in adolescents and young people.^13^ Furthermore, 25% of the mortality in anorexia nervosa is due to suicide.^4^

There is a current lack of understanding of the psychological, emotional and social factors that underpin the development and maintenance of SH across the ED diagnostic spectrum, with the management of low weight and associated risk often being the focus of attention in both research and treatment. While SH in young people without an ED is often understood to be a behavioural manifestation of being in acute psychological pain and/or a way of coping with or ameliorating this,^14 15^ it cannot be assumed to have the same function within the context of EDs. It is, therefore, not evident why a young person may develop both an ED and SH; furthermore, while SH may emerge before or after an ED, or simultaneous with it,^16^ their interrelationship and impact on one another are unknown.

These knowledge gaps have clear implications for treatment pathways and outcomes. Service provision and treatment options for young people with EDs alone are inconsistent across England.^17^ This is complicated by the fact that EDs are highly comorbid with other mental health problems, particularly SH and depression.^9 18^ When such comorbidities exist, referral pathways become unclear, which poses a significant challenge to providing optimal care in a timely manner.^17^ As such, NICE has called for the generation of evidence to understand the impact of comorbidities on treatment outcomes in EDs, explicitly mentioning SH.^19^

The ‘**S**elf-**H**arm **in E**ating Disorders’ (SHINE) study will explore the psychological, emotional and social factors that underpin the development and maintenance of SH in young people diagnosed with an ED. The study will (1) use ecological momentary assessment (EMA) to examine in real-time the psychological, emotional and social functions of SH in participants with ED (phase 1), (2) conduct in-depth qualitative interviews to gain participants’ perspectives on the genesis and function of their SH and ED, and their support needs (phase 2) and (3) work alongside young people with lived experience of SH and ED to explore the feasibility and acceptability of the study methods, disseminate findings and ensure that ethical standards are met.

### Objectives

Phase 1 will:

1. Examine the frequency, intensity and duration of SH thoughts and behaviours in young people with EDs, as they occur in real time.
2. Examine the context, function and processes surrounding SH thoughts and behaviours in young people with EDs.
3. Identify proximal factors predicting transition from SH thoughts to behaviours.
4. Explore the feasibility of recruiting for and conducting EMA studies with a sample of young people with an ED from three English outpatient services.

Phase 2 will:

1. Explore young people’s perspectives on the factors (social, cultural, experiential) that led to the development of their SH (thoughts and/or behaviours) and their ED.
2. Explore young people’s perspectives on the social and emotional functions of their SH and ED, and their inter-relationships.
3. Explore young people’s perspectives on the psychological/clinical/social support needed to address SH in EDs.

## Methods and analysis

### Study setting

SHINE is a multicentre study set in three specialist NHS outpatient ED services in England: (1) Forward Thinking Birmingham, Birmingham Women’s and Children’s NHS Foundation Trust, (2) Gloucestershire Health and Care NHS Foundation Trust and (3) Coventry and Warwickshire Partnership Trust. At all three sites, study activities will be the same, comprising recruitment of participants and data collection.

### Inclusion criteria

1. Aged 16–25.
2. ICD-10 diagnosis of ED (F.50.0 anorexia nervosa; F50.1 atypical anorexia nervosa; F50.2 bulimia nervosa; F50.3 atypical bulimia nervosa; F50.4 overeating associated with other psychological disturbances; F50.5 vomiting associated with other psychological disturbances; F50.8 other EDs; F50.9 ED, unspecified).
3. Current or previous SH thoughts and/or behaviours, in line with the NICE^1^ definition of SH as ‘An intentional act of self-poisoning or self-injury, irrespective of the motivation or apparent purpose of the act’.
4. Sufficient command of English.
5. Capacity to provide informed consent.

### Recruitment procedures

The study has been adopted as part of the NIHR portfolio and Clinical Research Network. Clinical Studies Officers (CSOs) (including assistant psychologists or research nurses depending on the trust), who are embedded with the care team at each of the three NHS Trusts, and who have the right to access service user data, will screen online medical notes/the trust’s databases (as appropriate to each trust’s systems) to identify eligible participants according to the inclusion criteria.

If those service users identified as potentially eligible are deemed by their care team (by Co-Is—authors SM, HB, and AW) or their own lead clinician if this is not one of the Co-Is) to have the capacity to consent and not to be too distressed to participate, they will be approached by their care team to inform them of the study and invite them to meet with the CSO, if interested. If the service user agrees, the CSO will contact them; provide the information sheet for phase 1; answer any questions they may have; and establish eligibility in relation to previous or current SH thoughts and/or behaviours using an adapted (shortened to 15-items) version of the Self-Injurious Thoughts and Behaviours Interview (SITBI).^20^ This will screen for history and/or the presence and frequency of thoughts and feelings of suicide and self-injurious behaviours.

If the service user screens positive on the adapted (shortened) version of the SITBI, the CSO will invite them to take part in phase 1 (EMA study) and provide them with at least 48 hours to consider their participation before informed consent is taken.

Participants will be requested to express whether they are interested in being reapproached for participation in phase 2 (Qualitative study) by initialling the relevant box on the phase 1 consent form.

In addition to the above, a poster will be placed in the waiting area of each outpatient service providing information about the study and encouraging participants to speak to their care team if they are interested in finding out more.

### Phase 1: an EMA study (objectives 1–4)

#### Participants

The EMA study sample will be a cohort of 70–100 young people (aged 16–25) with an ED diagnosis. The appropriate sample size was determined based on compliance rates of previous studies, whereby the population was young (16–25 years), mobile devices were used to prompt completion of a survey, and there was high sampling frequency (six prompts across 1 day). We will aim to recruit 100 participants aged 16–25 with an ED and SH thoughts or behaviours to take part in the EMA study; however, an acceptable number to be recruited will be 70 as per other EMA research studies. Thus, we will use 70 and 100 as our basis according to the compliance rates to ensure reasonable 95% CIs (see table 1).

**Table 1:** Three different compliance rates for sample sizes 70 and 100, which are deemed appropriate for this study

Sample size	Compliance rate	Study characteristic	Compliance rate proportion (95% CI)
70	90%	High sample frequency	0.90 (0.80 to 0.96)
70	80%	Younger people (<18 years old) / only mobile EMA platform	0.80 (0.69 to 0.89)
70	66%	Clinical studies with 4–5 frequency	0.66 (0.53 to 0.77)
100	90%	High sample frequency	0.90 (0.82 to 0.95)
100	80%	Younger people (<18 years old)/only mobile EMA platform	0.80 (0.71 to 0.87)
100	66%	Clinical studies with 4–5 frequency	0.66 (0.56 to 0.75)

Compliance rates with corresponding 95% CIs for sample sizes 70 and 100. Where we have an expected compliance rate of 66% for our sample size of 70, our 95% CI is 53% to 77%. Thus, we could be close to excluding many participants given an exclusion criterion of 50% compliance. In the cases where we have a sample size of 100, for all of the compliance rates, the 95% CIs become narrower.

EMA, ecological momentary assessment.

#### Procedure

After consenting, participants will complete the baseline questionnaire online using the Jisc Online Surveys platform. This questionnaire includes participant demographics; the Eating Disorder Examination Questionnaire 6.0 (EDE-Q),^21^ a 28-item scale measuring dietary restraint, eating concerns, concerns about weight and concerns about shape and a global score of ED, over the past 28 days on a 7-point Likert scale (0–6); the Clinical Impairment Assessment,^22^ a 16-item scale measuring ED-related psychosocial impairment over the past 28 days on a 4-point Likert scale (0–3) and the 72-item version of the SITBI to assess the presence, frequency and characteristics of SH thoughts and behaviours.^20^ Once the baseline questionnaire has been completed, each participant will be asked to download the Ilumivu mEMA app (https://ilumivu.com/) onto their mobile phone to capture EMA data in real time; this app has been used in other suicide and SH research.^23^ Participants will respond to a brief structured series of multiple-choice and free-text questions at each data entry point about the presence, form, context and functions of SH thoughts and/or behaviours, based on an adapted protocol used by Nock *et al*,^24^ and the 20-item version of the Positive and Negative Affect Scale (PANAS).^25^ Please see our online supplemental materials for the EMA questions. Data will be collected over 14 days. Data collection will involve quasi-random time-based responses, whereby six response prompts will be randomised within 4-hour time blocks between 9:00 and 21:00. This will give a representative sample of daily living and eliminate anticipatory effects. Participants will have 30 minutes from the prompt to begin completing the survey to ensure responses are contemporaneous.

10.1136/bmjopen-2022-065065.supp1 Supplementary data

### Statistical analysis

Data will be analysed using five strategies. First, we will use descriptive statistics to examine the demographics and baseline characteristics of the sample. Second, to assess study feasibility (objective 4), we will explore (1) the percentage of screened participants who were eligible (the eligibility rate), the percentage of invited participants who consented to participate (the consent rate), and whether there are any differences in demographic data between those screened who were ineligible versus those eligible and between those who consented versus those who were invited but declined to consent, (2) attrition rates (including missed surveys and study drop out) and whether there are any patterns in attrition (eg, whether the demographics of our sample predict EMA study drop out) and (3) the rates of ‘I don’t know’ and/or ‘Prefer not to say’ categories in our individual baseline and EMA surveys. The feasibility outcomes will be summarised and reported appropriately to help inform future studies. Third, we will examine the frequency, intensity, duration, co-occurrence, antecedents and consequences of SH thoughts and/or behaviours in young people with ED using descriptive statistics (objective 1). Fourth, descriptive statistics will be used to examine when thoughts and/or behaviour of SH occur in young people with ED (context); what young people are typically doing (function) and what they are feeling (PANAS positive and negative affect) (processes) (objective 2). Fifth, repeated measures logistic regression modelling will be used to test which contextual features of SH thoughts predict the occurrence (=1) or not (=0) of SH behaviours (objective 3). This multilevel modelling will account for the clustered nature of the observations, so observations within an individual participant within each day. The primary outcome (SH behaviour) specifically will be assessed at days 7 and 14. These study time points will be assessed to determine which time point (day 7 or day 14) is more informative for the primary outcome. We will report estimates of the outcome and corresponding 95% CIs by each group (young people with ED who have self-harmed vs young people with ED who have not self-harmed). Within the study design, every effort will be made to minimise missing data, and so the statistical analysis will be completed using complete case analysis. All statistical analyses will be completed using STATA (V.15 or above).

### Phase 2: in-depth qualitative semistructured interviews (objectives 5–7)

#### Participants

A purposive sample (according to sociodemographic variation, eg, age, gender and phenomenal variation, eg, ED diagnosis)^26^ of 20–30 young people who took part in phase 1 will be invited to take part in a single semistructured qualitative interview.

This sample size is appropriate to robustly capture a range of views and to anticipate reaching theoretical saturation.^27^ In keeping with the study design, we will use thematic analysis, for which 12 participants from a single group are required for saturation.^28^ However, given that the study is designed to explore participants’ experiences across demographic groups and ED diagnoses (some of which will overlap), we will recruit 20–30 participants, to ensure saturation across ED diagnoses.

#### Interview procedure

The interview study is underpinned by a medical anthropological approach, which comprises an interpretive framework focused on understanding young people’s lived experiences and the meanings they attribute to these.^29^ As such, the purpose of the interview will be to explore:

- Genesis: Why and when the participant’s SH (thoughts or behaviours) and ED developed, exploring the influence of social contexts and life events on each.
- Functions and inter-relationship: What functions the ED and SH each serve, and how these interrelate or may be different; and the factors influencing which behaviours are adopted, and in what circumstances.
- Support needs: What support (clinical, social, psychological) the participant feels they need in order to address SH in the context of an ED.

Interviews will be online, over a secure online platform (Microsoft Teams/Zoom). They will last approximately an hour, but the exact duration will be directed by participants. As in AL’s previous work on anorexia^30^ and SH,^31^ each interview will begin with the same open question: ‘If I say [the participant’s ED diagnosis] what do you think of?’ Fitting with the epistemological framework of the study, this approach is also crucial due to the lack of previous qualitative research on this topic. Moreover, allowing the participant to set the interview direction in this way signifies an ethical approach to a sensitive topic. It will maintain an openness to participants’ concerns throughout data collection, avoiding any assumptions on our part about relationships or disconnects between SH and ED. Each interview will also have space to reflect with participants on their own data from phase 1. We will explore motivations for a particular SH episode or thought recorded during the EMA study, discussing its emotional and social contexts as well as what the participant feels would have helped. We will also sensitively explore the reasons for any missing data in phase 1. Interviews will also give participants the opportunity to reflect on the experience of participating in the EMA study, which will inform future studies.

### Qualitative analysis

Interviews will be transcribed verbatim by a professional transcription company. Thematic analysis^32^ will include inductive and deductive coding. Inductive coding will ensure that the analysis is shaped by participants’ concerns and priorities. Deductive coding will allow us to systematically probe the data, asking questions arising from phase 1 and existing literature. Conducting the analysis concurrent with data collection will ensure iterative interaction between data and analysis to enhance reliability.^33^ We will also probe the relationship between each individual transcript and the themes across the interviews. Themes will be explored during this process within the study team and with the Young Persons’ Advisory Group (YAG).

### Data integration (phases 1 and 2)

Defined as ‘the interaction or conversation between the qualitative and quantitative components of a study’,^34^ data integration is key to mixed-methods research. The two data sets will be integrated in analysis using a mixed-methods matrix^35^ once data collection and preliminary analysis of each dataset is complete. A mixed-methods matrix is suitable due to the availability of both qualitative and quantitative data on all the participants who take part in phase 2.^34 36^ In the matrix, rows will represent the participants, with the columns detailing both types of data for each. We will look for comparisons and disconnects between the two types of data for a single participant as well as for patterns and anomalies across datasets. We will draw on the theoretical frameworks for SH^37^ (specifically, the integrated theoretical model of non-suicidal self-injury) and EDs^38^ (specifically, the cognitive-interpersonal maintenance model of EDs)—underpinning the study as well as the anthropological/sociological literature on both EDs^30 39^ and SH^40^ to interpret the findings.

We have preregistered our analyses for this study on the Open Science Framework: (https://osf.io/yqtrv/).

### Public and participant involvement

The study has been designed with members of the University of Birmingham Institute for Mental Health Young Persons’ Advisory group (YAG) of young people with lived experiences of mental health issues. Describing the study as ‘really needed’ and ‘ethical,’ YAG members were involved in the study from the outset, contributing to its overall design. The research question and objectives were informed by their priorities and they also advised the team on the appropriateness of methods; the burden of the intervention and time required to participate in the research; ethical considerations; and the feasibility of recruiting young people. Members’ involvement will be continued and invaluable. They will review emerging findings, be part of accessible dissemination and collaboratively set priorities for follow-on research. In addition to this wider involvement of YAG members, Co-I KR is an expert by experience and a member of the YAG. They co-designed the topic guide for the qualitative interview with the Principal Investigator (AL) and will be an equal member of the research team throughout the study.

## Ethics and dissemination

### Ethics

The study gained approval from the NHS HRA West Midlands—Black Country Research Ethics Committee (number: 296032). Although we recognise that it focuses on a sensitive topic and may pose some risks or burdens, we do not anticipate that taking part will adversely affect participants.

EMA has been used extensively in research with people with EDs^41 42^ and SH^43^ and has been found to be feasible and acceptable to participants, without any evidence of triggering symptoms of ED or SH. Compliance rates in studies with clinical populations are approximately 77%.^44^ This indicates that clinical populations, including young people, are willing and able to engage in EMA studies. In addition, previous research has demonstrated that engaging with SH and suicide research can be beneficial to participants and does not induce harm or increase risk.^45 46^ A recent meta-analysis demonstrated that engaging with suicide-related research actually reduced suicidal ideation, particularly within young people, and reduced the likelihood of engaging in suicidal behaviours.^47^

Qualitative interviewing has been used extensively with individuals with EDs^48^ and SH.^40^ Our previous work and other studies suggest that participants with SH^46^ or ED^30^ often welcome participation in qualitative interviews, finding the opportunity to reflect on their experiences beneficial. Crucially, a recent meta-analysis including both clinical and non-clinical populations indicated that being asked about suicidal experiences was not associated with an increase in distress and did not trigger or exacerbate suicidal thoughts or behaviours.^47^

The study Co-I at each site (SM, HB and AW) is a clinician within the trust’s ED service. This will ensure that the research is ethical, that any issues that may arise are dealt with appropriately, and that participants are safely supported throughout the research. A ‘Staff Information sheet’ will be given to the wider clinical care team of each participant, so that they are aware that their patient is taking part. This will also be sent to each participant’s GP by the CSO at the point of consent into phase 1. A copy of the consent form for each study phase will also be placed in each participant’s medical notes. The study also has a specific information sheet for relatives/friends which will be given to each participant on consent into the study. The aim of this is to aid participants to discuss the study with family members or friends should they wish to do so. Whether or not a participant passes on this information sheet is entirely their choice.

A Project Steering Committee of independent members including academics, young people with lived experience and clinicians will be set up at the outset and meet every 6 months to monitor study progress and ensure quality assurance and oversight.

The conduct of the research will also be monitored and audited on a monthly basis according to the research governance procedures of the Sponsor (the University of Birmingham).

### Dissemination

We anticipate the following outputs: (1) a Final Study Report in the NIHR Journals Library, (2) open access peer-reviewed publications and oral presentations at academic/public involvement conferences, (3) accessible findings (eg, infographics, podcasts) coproduced with YAG members for participants, practitioners and third-sector organisations. Participants will also receive an accessible report of the study results if requested during consent, (4) if they should like to, we will also apply with YAG members to be part of ESRC Festival of Science to enhance public understandings of SH and ED, (5) study website codesigned with YAG. In addition, to extend academic impact, we will also upload the anonymised qualitative data to the UK Data Archive (http://www.data-archive.ac.uk/), 24 months post-completion.

## Discussion

By addressing this underexplored topic through a novel mixed-methods design, this study will make a methodological contribution to the existing literature: it will assess the feasibility of combining EMA and qualitative interviews to understand the experiences, motivations and support needs of young people with complex mental health comorbidity. SHINE will also make a theoretical contribution: through its in-depth engagement with participants’ experiences, the study will establish why young people with ED may also self-harm. This knowledge will enable us to create a transdisciplinary theoretical model of the psychological, emotional and social factors that underlie SH in ED. The data will also establish participants’ social, psychological and clinical support needs. Given the high rates of SH in young people with an ED, and the current lack of treatment pathways, this model will be a crucial first step towards an empirical contribution: we aim that it will inform future NICE Guidelines and treatment interventions for addressing SH in ED and that it will potentially allow for greater flexibility and creativity in evidence-based pathway design or the restructure of existing services. In addition to these methodological, theoretical and empirical contributions, the study will also seek to enhance public understandings of SH and ED in young people, which is key to both reducing stigma and aiding help seeking. Central to this public engagement, and the study activities, as a whole, will be the ethical and valued input of young people with lived experience. As well as ensuring that the study design, praxis and directions are shaped by the priorities of young people, their involvement also has the aim of capacity building. Through it we seek to support young people with lived experience to become future leaders in applied mental health research.

## Supplementary Material

Reviewer comments
