# Supplementary material for: Self-Harm in Eating Disorders (SHINE): a mixed-methods exploratory study
Source: BMJ Open. 2022 Jul 27;12(7):e065065. doi: 10.1136/bmjopen-2022-065065 (PMC9335036; doi:10.1136/bmjopen-2022-065065)
Supplement: Supplementary data [file bmjopen-2022-065065supp001.pdf]

SHINE Study - Phase One EMA Protocol IRAS: 296032 (Version 2.1; 7th October 2021)

EMA PROTOCOL

1. **Presence of self-harm thoughts**

    a. *Since the last beep, have you thought about hurting yourself?*

If NO (ask the following and then go to question 6):

a. *What were you doing at the time of beep?* (free-text response)

b. *What were you feeling at the time of the beep*? (free-text response)

If YES, ask:

a. *Was it thoughts about direct self-harm i.e. harming yourself without wanting to die or attempted suicide i.e. harming yourself with the intention of dying. Select the behavior(s) you recently considered. You can click none, one, or both of these choices.*

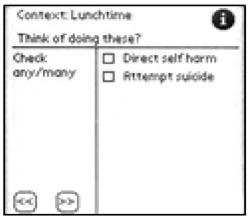

b. *Write down the method by which you considered harming yourself.* (free-text response).

2. **Context and characteristics of self-harm thoughts**

    a. *Indicate which activities you were doing while you were thinking about harming yourself. You can click on none, one, or more than one of these activities:*

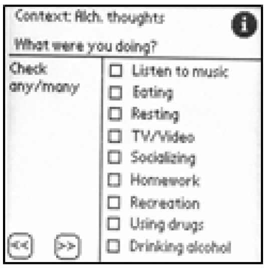

    b. *Indicate the person/people you were with while you were thinking about harming yourself. You can click on none, one, or more than one of these choices.*

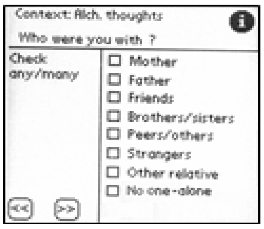

1

SHINE Study - Phase One EMA Protocol IRAS: 296032 (Version 2.1; 7th October 2021)

c. *Indicate things going on just before (and while) you thought about hurting yourself. If something led to the self-harm thoughts, but it is not listed, click on "Other" and another screen will appear for you to describe what it was in your own words. You can click on none, one, or more than one of these choices.*

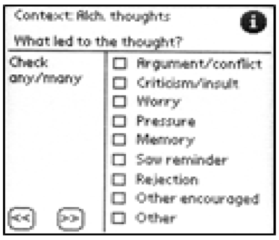

d. *Rate how intense the urge was to do the self-harming behavior (0 not present – 5 very severe). You are only permitted a single response here.*

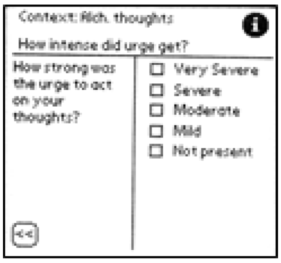

e. *Indicate when you first had the thought. The screen appears with the current time. If you are recording about thoughts you have now, this time is correct. If you are not recording about thoughts you have now, you will need to change this to the time when you first had the thought. Highlight the hours and/or the minutes numbers and then use the up and down arrows to switch the values of the numbers.*

3. Presence of self-harm behavior/ attempted suicide (depending on answer to question 1)

a. Did you harm yourself without wanting to die (direct self-harm) or with the intention of dying (attempted suicide).

0= self-harm
1= attempted suicide

b. *What did you do to hurt yourself?* (free-text response)

4. Context and characteristics of self-harm behaviour

a. *Indicate how long you thought about doing the behavior you selected above. This is the time spent thinking about doing it since the time it first occurred to you until the time you did it or were able to think about other things. Click on a time interval. You are only permitted a single response here.*

Lavis A, *et al. BMJ Open* 2022; 12:e065065. doi: 10.1136/bmjopen-2022-065065

SHINE Study - Phase One EMA Protocol IRAS: 296032 (Version 2.1; 7th October 2021)

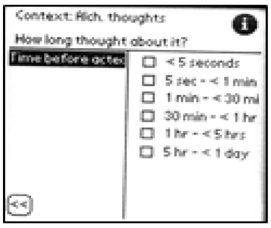

b. *Indicate what you were feeling when you had the thought(s)/behavior(s) you selected above. Click on none, one, or more than one of these feelings.*

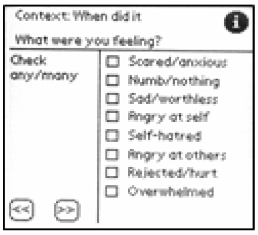

c. *Indicate how long you spent doing the behavior you selected above. Click on the time interval. You are only permitted a single response here.*

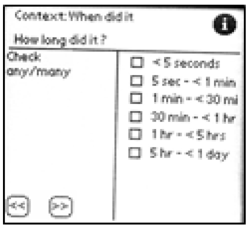

5. **Functions of self-harm behavior/attempted suicide (depending on answer to question 1)**
   a. *Indicate why you did the behavior. If the first four are not right, click on "Other". Click on none, one, or more than one of these reasons.*

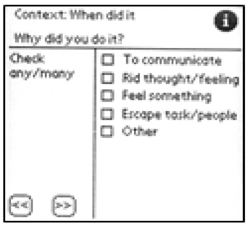

[If "*To communicate*" was selected, move to question 5b. If "Rid thoughts/feelings was selected, skip to question 5e. If "feel something" was selected, skip to question 5g. If "escape task/people was selected, skip to question 5i]

   b. *Specify what you were trying to communicate or what impact you were trying to get when you did the behavior. Click on none, one, or more than one.*

Lavis A, *et al. BMJ Open* 2022; 12:e065065. doi: 10.1136/bmjopen-2022-065065

SHINE Study - Phase One EMA Protocol IRAS: 296032 (Version 2.1; 7th October 2021)

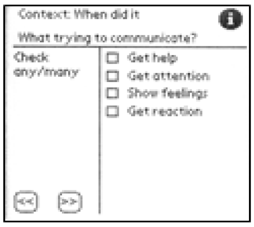

c. Identify the people from whom you got a response to your behavior. Click on none, one, or more than one.

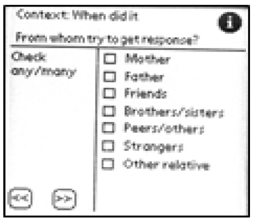

d. Report the results of your attempts to communicate by doing the behavior you did. Click on none, one, or more than one.

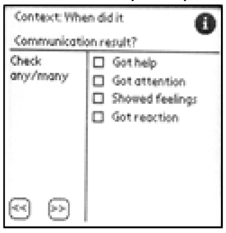

e. Indicate the feeling(s)/thought(s) you were trying to relieve by doing the behavior you did. Click on none, one, or more than one.

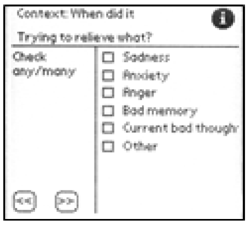

f. Indicate the feeling(s)/thought(s) that were relieved by doing the behavior you did. Click on none, one, or more than one.

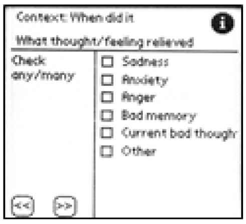

SHINE Study - Phase One EMA Protocol IRAS: 296032 (Version 2.1; 7th October 2021)

g. *Indicate what you were trying to feel by doing the behavior you did. Click on none, one, or more than one.*

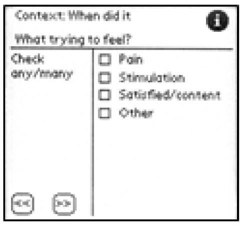

h. *Indicate what you felt when you hurt yourself. Click on none, one, or more than one.*

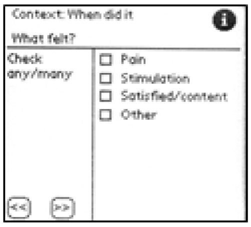

i. *Indicate what you were trying to escape by doing the behavior you did. Click on none, one, or more than one.*

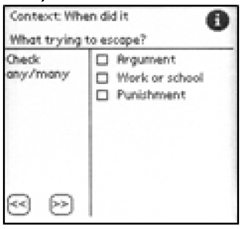

j. *Indicate from whom you were trying to escape by doing the behavior you did. Click on none, one, or more than one.*

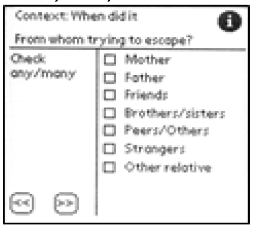

6. Alternative behavior

a. *Identify the other activities you did instead of hurting yourself. If you did something else instead, but it is not listed, click on "Other" and another screen will appear for you to describe what you did in your own words. Click none, one, or more than one of the choices offered.*

Lavis A, *et al. BMJ Open* 2022; 12:e065065. doi: 10.1136/bmjopen-2022-065065

SHINE Study - Phase One EMA Protocol IRAS: 296032 (Version 2.1; 7th October 2021)

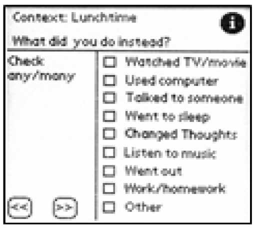

7. **Positive and negative affect** using the 20-item short-form of the Positive and Negative Affect Scale (PANAS; Watson et al., 1988). Questions are rated on 1–5 rating scale (1 very slightly or not at all to 5 extremely). The total scores for positive affect and negative affect are calculated separately by summing the ratings on each scale.

At the end of the log we have given you an opportunity to tell us about something else that might have happened.
