# Supplementary material for: Self-Harm in Eating Disorders (SHINE): a mixed-methods exploratory study
Source: BMJ Open. 2022 Jul 27;12(7):e065065. doi: 10.1136/bmjopen-2022-065065 (PMC9335036; doi:10.1136/bmjopen-2022-065065)
Supplement: Reviewer comments [file bmjopen-2022-065065.reviewer_comments.pdf]

PEER REVIEW HISTORY

BMJ Open publishes all reviews undertaken for accepted manuscripts. Reviewers are asked to complete a checklist review form (**http://bmjopen.bmj.com/site/about/resources/checklist.pdf**) and are provided with free text boxes to elaborate on their assessment. These free text comments are reproduced below.

ARTICLE DETAILS

**TITLE (PROVISIONAL)**	Self-Harm In Eating Disorders (SHINE): A Mixed-Methods Exploratory Study
**AUTHORS**	Lavis, Anna; McNeil, Sheryllin; Bould, Helen; Winston, Anthony; Reid, Kalen; Easter, Christina; Pendrous, Rosina; Michail, Maria

This article was not externally reviewed at BMJ Open. Protocols that have been independently assessed prior to submission to BMJ Open are usually fast-tracked to publication on the grounds that further substantial changes will not be possible. This independent assessment will usually be external, independent review for both a substantial grant award from a non-commercial or government funder and ethics approval.
